# Benign acute childhood myositis: a scoping review of clinical presentation and viral etiology

**DOI:** 10.1007/s00431-024-05786-y

**Published:** 2024-09-25

**Authors:** Elli Majava, Marjo Renko, Ilari Kuitunen

**Affiliations:** 1 Kuopio Pediatric Research Unit, University of Eastern Finland, Kuopio, Finland; 2 Department of Pediatrics and Neonatology, Kuopio University Hospital, Puijonlaaksontie 2, Uusi Sydän 8Krs, 70211 Kuopio, Finland

**Keywords:** Myositis, Infectious diseases, Limping, Influenza, Viral infection

## Abstract

Benign acute childhood myositis is a benign phenomenon often associated with influenza in children. The aim of this scoping review was to analyze the typical clinical picture and characteristics of benign acute childhood myositis patients. Furthermore, we aimed to analyze the epidemiology and viral findings. We performed a systematic scoping review. We searched PubMed, Scopus, Web of Science, and CINAHL databases in August 2023. We included observational studies that focused on children and reported at least 10 children. Our main outcome was to describe the typical clinical picture of benign acute childhood myositis patients. Furthermore, we aimed to report the typical laboratory findings and virus findings. A total of 211 studies were screened, and 22 studies were included in this review. The included studies were mainly from Europe (13 studies) and Asia (5 studies). Two studies were prospective, and the rest were retrospective. The mean age of the patients was 6.8 (CI 5.8–7.8) years. Benign acute childhood myositis appeared to be more prevalent among boys in all studies. The most prevalent symptoms were bilateral leg pain (pooled prevalence 92%), followed by fever (80%) and inability to walk (56%). Hospitalization rates varied between 4 and 100%, and the mean hospital stay was 3.6 (CI 3.3–3.9) days. Influenza B was the most common virus detected, followed by influenza A. Other reported viruses included herpes simplex, coxsackie-, enteroviruses, adeno-, respiratory syncytial, and parainfluenza viruses. All studies reported creatinine kinase levels, and the reported mean values varied between 100 and 4000 U/L, whereas only five studies reported C-reactive protein which was only slightly elevated.

*Conclusion:* According to a systematic assessment of published literature, benign acute childhood myositis patients were typically school-aged children, presenting with bilateral leg pain, fever, and inability to walk. Influenza A and B were the most reported viruses, but multiple other viruses have been associated with benign acute childhood myositis. These patients have high creatinine kinase values, but their hospital stay was rather short. Overall, this review provides important information for clinicians on the characteristic presentation of benign acute childhood myositis, and these findings may help to better identify these patients and reduce unnecessary tests.

**Table:** 

**What is known:** *• Benign acute childhood myositis has been mainly associated with influenza viruses, especially B influenza.* *• Typical patients have been reported to be under school-aged children, and boys have been reported to have a higher incidence.*	
**What is new:** *• Many non-influenza viruses were also associated with benign acute childhood myositis, such as herpes simplex, coxsackie-, entero-, adeno-, respiratory syncytial, and parainfluenza viruses.* *• Typical symptoms are bilateral calf pain, fever, and inability to walk and creatine kinase levels were reposted to be increased.*

**Supplementary Information:**

The online version contains supplementary material available at 10.1007/s00431-024-05786-y.

## Introduction

Benign acute childhood myositis (BACM) is a syndrome that typically occurs after an upper respiratory illness, causing pain in both legs. The first study reporting on it was published in Sweden in 1957 by Lundberg and discussed 74 cases of calf myalgia [1]. BACM is primarily seen in preschool-aged children, and it has been reported to be more common in boys than girls [2, 3]. The incidence of BACM cases seems to follow the peaks of seasonal influenza [9], but also other viruses have been presented as etiological agents [2–8].

Typical BACM patients have first had fever and other upper respiratory tract infection symptoms. After a few days, severe bilateral pain in the legs, especially in calves, emerges. The pain often leads to walking difficulties and even the inability to walk [4]. Increased CPK (creatine phosphokinase) levels in serum have been reported in many studies among BACM patients [2, 5, 6]. The mean time between the onset of fever and the onset of calf pain is 3 days [7]. The typical recovery period has been described to be short, lasting around 4 days [3, 7]. The diagnosis of the disease can be challenging, as the intense symptoms may result in consideration of more serious conditions, such as rhabdomyolysis or polyneuropathies. This may lead to unnecessary examinations and raise concerns in patients and parents.

In this scoping review, we aimed to gather more information about the clinical presentation of BACM. Furthermore, we aimed to comprehensively investigate the laboratory findings of BACM patients and which viruses have been associated with BACM in children.

## Methods

We conducted a scoping review and reported our review according to the Preferred Reporting Items in Systematic Reviews and Meta-analyses extension of Scoping Review (PRISMA-ScR) guideline [8].

### Search strategy

We searched PubMed, Scopus, Web of Science, and CINAHL Complete (Ebsco) databases on August 31st, 2023 from inception. The search string was acute AND benign AND myositis. We did not use any filters in the search process. Search results were then uploaded to Covidence software where duplicates were first automatically removed. Two authors independently screened the abstracts and later full reports against the eligibility criteria. Disagreements were solved by reaching a mutual consensus.

### Inclusion and exclusion criteria

The included studies had to focus on children (aged 0 to 18 years) and describe symptoms of acute benign myositis. We intended to include both prospective and retrospective studies and interventional as well as observational studies. We did not pre-specify the outcomes, as our aim was to gather an overview of the current literature. We did however exclude case studies and case series where less than 10 children were included, as these are most likely highly selected examples. We excluded studies that were not reported in English. Furthermore, studies that did not report any original data (reviews, editorials, etc.) were excluded.

### Aims

Our main aim was to describe the characteristic clinical presentation of BACM patients. Furthermore, we aimed to report the typical laboratory findings and microbiological findings of BACM patients. Finally, we aimed to report the incidence of BACM.

### Data extraction

One author extracted the data to a pre-designed Excel spreadsheet, and another author validated the data to reduce potential errors during the extraction process. We extracted the following information from each included study: authors, journal, country, study period, patient characteristics, BACM definition, virus findings, laboratory findings, and conflict of interest.

### Statistics

To uniform the reporting, we have converted all reported continuous variables into means and standard deviations. We have pooled findings by using the Mantel–Haenszel method for categorized outcomes and inverse variance meta-analysis for continuous outcomes. We have presented the pooled prevalences for symptoms and the pooled means for patient age, hospitalization duration, and time to recovery with 95% confidence intervals (CI).

### Protocol registration

Protocol for this scoping review was registered to Open Science Framework, 10.17605/OSF.IO/KYCBT, and the protocol is available from: https://osf.io/kycbt/.

## Results

**Fig. 1:**
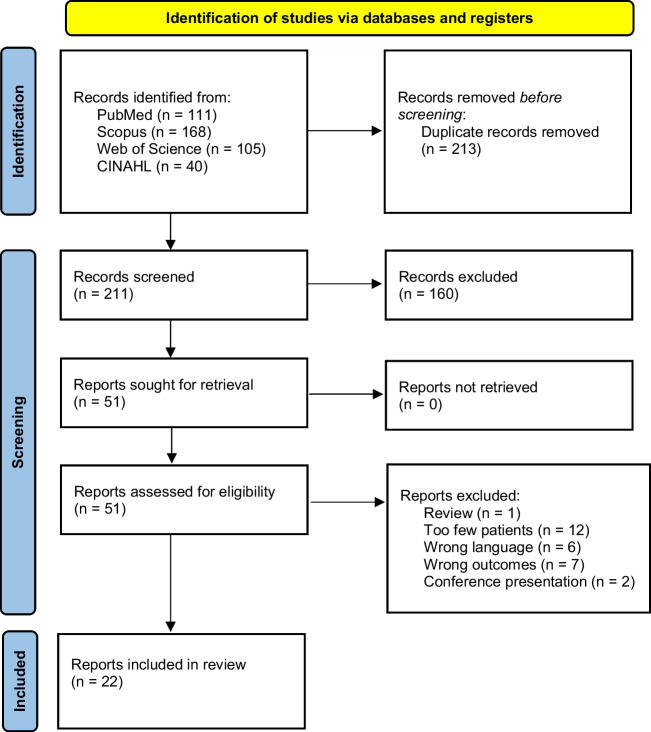
Prisma flowchart of the study selection process

We identified 211 unique studies, and after screening the abstracts, we assessed 51 full reports. Finally, we included 22 studies (Fig. 1) [2–7, 9, 10]. The included studies were conducted mainly in Europe (13 studies), and in Asia (5 studies), and the number of the included pediatric patients varied between 10 and 219 (Table 1). All studies were observational, only two were prospective, and the remaining 20 studies were retrospective. The most commonly used main outcomes were the clinical presentation of the patients, followed by viral etiology and other laboratory findings (Table 1). The extracted data is uploaded to supplementary materials.

**Table 1:** Characteristics of the included studies

Study	Country	Study period	Retrospective or prospective	*N* of patients
Al-Qahtani et al. 2015 [6]	Saudi Arabia	2008–2012	Retrospective	28
Attaianese et al. 2023 [2]	Italy	2018–2023	Retrospective	65
Brisca et al. 2021 [5]	Italy	2010–2018	Retrospective	113
Costa Azevedo et al. 2022 [4]	Portugal	2015–2019	Retrospective	100
D’Amico et al. 2022 [15]	Italy	2008–2020	Retrospective	50
Ferrarini et al. 2014 [3]	Switzerland	1/2013–4/2013	Prospective	49
Karpathios et al. 1995 [16]	Greece	2/1991–4/1991	Retrospective	12
Kerr et al. 2021 [17]	Australia	2011–2015	Retrospective	226
Mackay et al. 1999 [9]	Australia	1978–1997	Retrospective	38
Mall et al. 2011 [7]	Germany	2007–2008	Retrospective and prospective	219
Rajajee et al. 2005 [13]	India	2001–2002	Retrospective	40
Rosenberg et al. 2018 [18]	Israel	2010–2013	Retrospective	54
Ruff and Secrist 1982 [19]	United States	1977–1999	Retrospective	35
Saltık et al. 2012 [20]	Turkey	1/2011–3/2011	Retrospective	15
Sham 2015 [21]	Hong Kong	2003–2012	Retrospective	36
Szenborn et al. 2018 [22]	Poland	2012–2015	Retrospective	13
Tekin and Akoğlu 2022 [23]	Turkey	2019–2020	Retrospective	25
Turan et al. 2022 [12]	Turkey	2016–2019	Retrospective	114
Yoon et al. 2018 [14]	Korea	2010–2016	Retrospective	47
Yorulmaz et al. 2019 [24]	Turkey	2017–2018	Retrospective	22
Zafeiriou et al. 2000 [10]	Greece	1996–1999	Retrospective	32
Öztürk et al. 2022 [11]	Turkey	2018–2020	Retrospective	94
Total			2 prospective, 22 retrospective	1427

The mean age of the patients was 6.8 years (CI 5.8–7.8). BACM was more prevalent among boys than in girls in all studies (Table 2). The majority of patients experienced bilateral calf pain (pooled prevalence 92%) with over half of the studies indicating a 100% incidence of this symptom. The inability to walk was reported by 56% of the patients. Fever was also a common symptom, and it was reported to be present between 40 and 100% of the patients, with a pooled prevalence of 80% (Table 2). Symptoms resulted in at least some hospitalizations in almost every study we included, although the proportion of admissions varied from 4 to 100% (Table 2). The mean duration of hospitalization was 3.6 days (CI 3.3–3.9), and the recovery time ranged from 1.3 to 6.3 days.

**Table 2:** Patient characteristics in the included studies

Study	Age (years, mean ± SD)	Sex	Muscle symptoms		Proportion of patients with fever	Inpatient admission	Length of hospital stay (days, mean ± SD)	Time to recovery (days, mean ± SD)
		Girls/Boys	Bilateral leg pain	Inability to walk				
Al-Qahtani et al. 2015	6.3 ± 7.0	4/24	100%	93.3%	100%			
Attaianese et al. 2023	6.3 ± 2.2	22/43	100%	93.8%	75.3%	24%	5.1 ± 3.5	2.9 ± 1.7
Brisca et al. 2021	6.8 ± 2.2	28/85	92%	77%	85%	85%	4.8 ± 1.8	
Costa Azevedo et al. 2022	6.7 ± 3.0	23/77	92%	29%	87%	29%	2 ± 1.6	
D’Amico et al. 2022	5.4 ± 2.4	7/43	100%	38%	84%	100%	4.9 ± 2	
Ferrarini et al. 2014	7.4 ± 2.5	18/31	100%	4% (full)	57%	4%	< 3	4 ± 1.5
Karpathios et al. 1995	4.5–11	2/10	100%	83.3%	100%	100%		4–5
Kerr et al. 2021	6.0 ± 1.5	41/185	70%			19%	1.7 ± 1.5	
Mackay et al. 1999	8.1 ± 2.5	6/32	78%	19%	80%			7
Mall et al. 2011	7.3 ± 2.2	59/160	100%		100%	47%		3.7 ± 1.5
Rajajee et al. 2005	5.3	18/22	85%	45%				< 7
Rosenberg et al. 2018	7.3 ± 1.9	11/43	81%		72%	74%	3.2	
Ruff and Secrist 1982	4–15	15/20	100%		100%			4–10
Saltık et al. 2012	6.3 ± 2.1	3/12	100%	93.3%	93.3%	100%		3
Sham et al. 2015	7.8 ± 3.1	9/27	100%		100%	100%	3 (median)	
Szenborn et al. 2018	6.5 ± 3.0	5/8	62%	46%	100%	100%	2 (median)	3 (median)
Tekin and Akoğlu 2022	6.9 ± 3.6	4/21	Majority	20%	44%	66%	3.3 ± 2.9	2–3
Turan et al. 2022	8.1 ± 3.1	24/90	91.2%	29.8%	45.60%	21.1%		5.8 ± 1.4
Yoon et al. 2018	6.8 ± 3.1	9/38	100%		100%	100%	5.7 ± 2.3	4.2 ± 1.3
Yorulmaz et al. 2019	7.1 ± 2.2	6/16	100%	54.5%	81.8%	45.5%	6.3 ± 1.8	< 3
Zafeiriou et al. 2000	7.3 ± 2.1	14/18	93.8%	59.4%	40.6%	31.2%		2–7
Öztürk et al. 2022	6.4 ± 1.8	27/67	100%	100%	64%	100%	1.3 ± 1.2	3.2 ± 1.4
Studies that reported information (*N*)	22	22	22	16	20	18	13	15
Total*	6.8 (CI 5.8–7.8)	355/1072	92% (CI 86–98%)	56% (CI 53–59%)	80% (CI 77–82%)	51% (CI 46–57%)	3.1 (CI 2.0–4.2)	3.6 (CI 3.3–3.9)

^*^Weighted proportions with 95% confidence intervals, pooled together by the Mantel–Haenszel meta-analysis method. The inverse variance method used for the pooling of means

Viral etiology was reported in 16 studies. These studies indicated that influenza B was the most common virus detected among BACM patients. Influenza A was also a common finding while Herpes simplex, coxsackie-, entero-, adeno-, respiratory syncytial, and parainfluenza viruses, and a few other viruses were less commonly identified (Supplementary Table 1).

All 23 studies reported CPK levels. The mean values ranged between 1185 and 14,319 U/L (Supplementary Table 2). The normal range for CPK varies between 40 and 240 depending on the used laboratory technique [9]. Alanine aminotransferase (ALAT/ALT) values were also reported in seven studies, the mean values varying between 31 and 150 U/L (Supplementary Table 2). Finally, C-reactive protein (CRP) was evaluated in five studies, and the measured values were slightly increased.

## Discussion

In this scoping review, we found that practically all children having BACM suffer from bilateral leg pain and almost all have fever. The inability to walk was a common symptom although its prevalence varied from 4 to 100%. Bilateral calf pain and inability to walk are rather dramatic clinical symptoms which should rapidly guide the clinicians’ diagnostic process toward BACM. Elevated CPK was the most characteristic laboratory finding in BACM patients. To date, this review is the largest performed on the clinical presentation and laboratory findings of BACM.

The proportion of patients needing inpatient admission varied widely in the included studies. This was probably due to different study settings and case definitions as some of the studies focused on hospitalized patients only, whereas some included all patients with calf pain in the emergency department. This variability may also be attributed to local differences, as the criteria for hospitalization can be quite subjective in cases of benign conditions that are, however, characterized by intense pain. The duration of the hospital stay was mostly between 3 and 5 days, which was rather short as the majority of the patients reported to have totally recovered within a week. BACM recurrence is rare. Öztürk et al. reported no recurrence [11]. In one of the studies, recurrences were carefully examined and found to occur in 9.8% of the 113 cases. One patient experienced BACM three times in 3 years [5].

Currently, the recommended treatments for BACM are good hydration and to follow urine output. Symptomatic analgesia is indicated as needed. The symptoms resolve without specific medical treatment [2]. Turan et al. investigated whether oseltamivir treatment affects the recovery time of patients with BACM. Their study showed that those who received oseltamivir during treatment recovered in a median of 4 days, while those who did not receive it recovered in a median of 5 days [12]. Therefore, the benefit of the oseltamivir treatment appeared to be minimal clinically, but it was statistically significant. A common practice has been to monitor urine output in at least hospitalized BACM patients. Despite the high CPK levels, no cases of myoglobinuria were observed in previous reports [5]. In the included studies, renal failure was very rare as only one study by Turan et al. reported two cases [12]. As the majority of BACM patients did not have renal issues, it seems rather safe to treat these patients at home if hydration is possible.

A higher proportion of male patients was observed in all articles. However, none of them provided explanations for this observation. Mackay et al. suggested in 1997 [9] that the reason could be a genetic predisposition or an unknown metabolic cause, to which other articles have mainly referred. Additionally, Rajajee et al. [13] mentioned that the reason could be the higher physical activity level among boys. BACM predominantly affects children aged 5 to 9 years old and is rarely diagnosed in adults, which is an interesting observation. The precise mechanism is unclear and needs more research to uncover causes and contributing factors. A high physical activity with a reluctance to rest may contribute to this condition. Despite the resolution of the respiratory infection, the causative virus may persist in the body. Physical activities such as walking, running, and playing might lead to the development of myositis in the calves. The muscles of children in this age group are still in the developmental stage, with longitudinal growth actively ongoing.

The majority of the studies have focused on influenza, particularly on influenza B as an etiology for BACM [11, 14]. However, this review highlighted a wide variety of viruses among BACM patients (Table 2). Also, in the study of Yoon et al., the “others” category accounted for nearly one-quarter of cases. Moreover, the reliability of the microbiological results may be limited, as many studies solely focused on testing for influenza and the proportion of tested patients was low in most studies. Testing was usually made with nose/throat swabs and then examined by real-time reverse transcription polymerase chain reaction. Some physicians tested nose/throat swabs by rapid test in their own practice [14]. Previous studies have reported that the viral etiology remained unknown despite testing, with rates varying between 5 and 66% [15]. Thus, it is important to recognize the role of non-influenza viruses as potential additional causative agents of BACM. Although most cases occur during the annual influenza epidemic peaks, it is possible to encounter BACM throughout the year, as the alternative viruses include viruses without clear epidemic patterns.

Creatine phosphokinase is an enzyme released from muscle cells in response to muscle damage. Elevated concentration of CPK is the most prominent laboratory finding in BACM patients. CPK levels were reported to reach up to 54,000 U/L, but the cases had good clinical outcomes and recovered without complications. All cases had a benign course. Elevated levels of CPK can be considered a useful tool in diagnostic evaluation, and its decrease correlates with the healing process.

The main strength of this review was the comprehensive search strategy and systematic assessment of the literature against clear inclusion and exclusion criteria. Furthermore, this is the largest review on the topic as far as we are aware. The main limitation is the high heterogeneity in the inclusion criteria and study settings of the included studies. Due to these factors, we did not perform any statistical synthesis. Additionally, we were unable to report the incidence of BACM as the studies were not population-based assessments.

## Conclusion

According to this systematic assessment of published literature, BACM patients were typically school-aged children, and bilateral calf pain, fever, and inability to walk were the most prevalent symptoms. Influenza A and B were the most frequently reported viruses, but multiple other viruses were associated with BACM. BACM patients had high creatinine kinase values, but kidney involvement was exquisitely rare, and hospitalization was usually rather short. Recognizing the typical clinical picture of BACM may help clinicians to better identify BACM patients and reduce unnecessary tests and examinations.

## Supplementary Information

Below is the link to the electronic supplementary material.

Supplementary file1 (DOCX 35 KB)

## Data Availability

All data available upon request from the corresponding author.
